# Association of tumor locations, clinical manifestations, and histopathological grade in meningioma: a 10-year single-center study

**DOI:** 10.3389/fonc.2026.1871594

**Published:** 2026-07-28

**Authors:** Tiara Aninditha, David Tandian, Rini Andriani, Henry Riyanto Sofyan, Irma Savitri, Adrian Ridski Harsono, Jonathan Odilo

**Affiliations:** 1 Department of Neurology, Dr. Cipto Mangunkusumo National General Hospital, Faculty of Medicine, Universitas Indonesia, Jakarta, Indonesia; 2 Department of Neurosurgery, Dr. Cipto Mangunkusumo National General Hospital, Faculty of Medicine, Universitas Indonesia, Jakarta, Indonesia; 3 Department of Neurology, Dharmais National Cancer Center, Jakarta, Indonesia; 4 Department of Neurology, Universitas Indonesia Hospital, Depok, Indonesia

**Keywords:** clinical manifestations, convexity, cranial base, Indonesia, meningioma, neuro-oncology, tumor location, World Health Organization grade

## Abstract

**Background:**

Meningiomas are the most common primary intracranial tumors, with clinical manifestations that vary depending on tumor location and biological behavior. Understanding the relationship between tumor location, presenting symptoms, and histopathological grade is essential for improving diagnostic accuracy and clinical management. This study aimed to evaluate the association between tumor location, clinical manifestations, and World Health Organization (WHO) grade in patients with intracranial meningioma.

**Methods:**

This retrospective observational study included consecutive patients aged ≥18 years who underwent first-time intracranial meningioma resection over a 10-year period (2013–2023) at Cipto Mangunkusumo National General Hospital, Jakarta, Indonesia. Demographic data, presenting symptoms, tumor location (cranial base vs. non–skull base, with convexity, parasagittal, falcine, and intraventricular tumors grouped as the non–skull base/convexity category), Simpson grade of resection (I–V), and WHO histopathological grade were extracted from medical records. Pearson’s chi-square test was used to assess associations between categorical variables and tumor location.

**Results:**

A total of 531 patients were included. The majority were female (92.1%) with a median age of 45 years (range 18–82). Cranial base meningiomas accounted for 77.2% of cases. Female gender (p=0.037), age <60 years (p<0.001), WHO grade I tumor (p<0.001), a higher proportion of Simpson grade III–V resection (p<0.001), cranial nerve deficits (p<0.001), and visual field deficits (p=0.010) were significantly more frequent in cranial base meningiomas. In contrast, decreased consciousness (p<0.001), headache (p=0.023), motor deficits (p<0.001), sensory deficits (p=0.009), and seizures (p<0.001) were significantly more frequent in non–skull base/convexity meningiomas.

**Conclusion:**

Tumor location in meningioma is associated with specific clinical manifestations and histopathological grade. Cranial base tumors tend to present with cranial nerve–related and visual symptoms, whereas non–skull base tumors are more likely to cause seizures, motor deficits, and decreased consciousness. Higher-grade tumors are more commonly found in non–skull base locations. The disproportionately high female-to-male ratio observed in this Indonesian surgical cohort should be interpreted with caution, as selection and referral bias may have contributed; it warrants confirmation in population-based studies that include molecular profiling and prospectively collected hormonal-exposure data.

## 1 Introduction

Meningioma is the most common primary brain tumor and originates from arachnoid cap cells. It accounts for approximately 56.2% of all non-malignant central nervous system tumors, with an annual incidence of 9.73 per 100,000 population (1). Meningioma incidence is significantly higher in females than in males (2.4:1), and more than 95% of cases are classified as World Health Organization (WHO) grade 1 or benign (1, 2).

The mass effect of meningioma produces a wide variety of symptoms depending on the compressed structures involved. Schankin et al. found that 85% of patients with brain tumors experienced secondary headache, with meningioma being significantly associated with secondary headache (3). Seizures, caused by changes in the local microenvironment and vasogenic edema surrounding the tumor, occur in 20–50% of meningioma patients (4). Focal neurological deficits, including motor, sensory, and cranial nerve deficits, are also common; Meling et al. reported focal neurological deficits in up to 57% of meningioma patients (5).

The anatomical distribution of meningiomas has been well investigated. Sun et al. found that meningiomas most commonly occur on the convexity (20.8%), parasagittal region (16.1%), and falx (11.4%), and less frequently at cranial base sites such as the sphenoid (9.8%), cerebellar tentorium (8.5%), and cerebellopontine angle (7.7%) (6). Hashimoto et al. demonstrated that cranial base meningiomas have slower growth and lower MIB-1 indices compared with non–skull base meningiomas (7). Maiuri et al. reported that progesterone receptor (PR) expression in meningioma is more abundant in cranial base than in other locations (8). Apra et al. observed that women with skull base meningioma have higher progesterone receptor (PR) expression than men (9), and grade 2 meningiomas are more commonly found outside the skull base (10).

Despite these epidemiological insights, large-scale data on the relationship between tumor location, presenting symptoms, and WHO grade in Southeast Asian populations remain limited. Presenting symptoms vary widely among meningioma patients and influence the choice of therapeutic approach. The aim of this study was to describe and analyze the presenting symptoms of intracranial meningioma in relation to demographic characteristics, tumor location, and WHO grade in patients who underwent surgery between 2013 and 2023 at Cipto Mangunkusumo National General Hospital.

## 2 Materials and methods

### 2.1 Study design and setting

This was a single-center retrospective observational study based on the medical record database of Cipto Mangunkusumo National General Hospital, Jakarta, Indonesia. The study was conducted and reported in accordance with the Strengthening the Reporting of Observational Studies in Epidemiology (STROBE) guidelines for observational studies.

### 2.2 Patient enrollment

Consecutive patients aged 18 years or older who underwent first-time intracranial meningioma resection between 2013 and 2023 were enrolled. Patients with previously treated meningioma or incomplete medical records were excluded. A total of 531 patients met the inclusion criteria and were included in the analysis. As only surgically treated patients were eligible, meningioma cases managed conservatively (e.g., small, asymptomatic incidental tumors) were not represented in the cohort; this selection issue is addressed in the Discussion.

### 2.3 Variables and data collection

Demographic variables (age and sex), WHO histopathological grade, tumor location (cranial base vs. convexity, with further classification by anatomical sub-site), Simpson grade of resection, and presenting clinical signs and symptoms were extracted from medical records. Tumor location was classified anatomically into two principal categories. Cranial base meningiomas were defined as tumors arising from any portion of the anterior, middle, or posterior cranial fossa floor, including sphenoorbital, sella–suprasellar (including clinoidal), olfactory groove, cavernous sinus, petroclival, cerebellopontine angle, posterior fossa (tentorial and infratentorial), basal frontal and temporal, and orbital/retro-orbital tumors. The non–skull base/convexity category included tumors arising from the inner surface of the calvarium (true convexity), parasagittal region, falx (falcine meningiomas), and intraventricular spaces. Pineal-region meningiomas were classified as non–skull base because of their supratentorial, deep midline location. Each case was classified by review of the preoperative MRI report and confirmed against the intraoperative surgical record by two investigators; discrepancies were resolved by consensus.

Histopathological grading was performed by experienced neuropathologists according to the World Health Organization (WHO) Classification of Tumors of the Central Nervous System applicable at the time of original diagnosis (2007, 2016, and 2021 editions). To ensure consistency across the 10-year study period, cases diagnosed before 2016 were re-classified retrospectively using the 2016 WHO criteria, and the small number of cases reclassified upward (n<5) is reported in the Results. Cases diagnosed between 2017 and 2021 followed the 2016 criteria; cases diagnosed in 2022 or later followed the 2021 WHO criteria. WHO grades II and III were combined for analysis owing to the small number of grade III cases.

Simpson grade of resection was recorded as originally documented by the operating neurosurgeon (grades I through V): grade I, macroscopically complete removal with excision of dural attachment and any abnormal bone; grade II, macroscopically complete removal with coagulation of dural attachment; grade III, macroscopically complete removal without resection or coagulation of dural attachment; grade IV, partial removal leaving tumor *in situ*; and grade V, simple decompression with or without biopsy.

Clinical variables were defined as follows: decreased consciousness, any reduction in Glasgow Coma Scale score below 15 documented at admission; headache, persistent or recurrent headache attributable to the tumor as documented in the neurology referral note; cranial nerve deficit, any documented dysfunction of cranial nerves III–XII established by standard bedside neurological examination performed by the attending neurologist (impairments of optic nerve fundus appearance and formal visual field assessment were recorded separately as ‘optic nerve abnormality’ and ‘visual field deficit’, respectively, so that they could be analyzed independently of motor/sensory cranial nerve deficits); motor deficit, any documented weakness with Medical Research Council grade <5/5 on neurological examination; sensory deficit, any documented hemisensory or focal sensory loss; seizure, at least one witnessed or self-reported seizure attributable to the tumor prior to surgery; visual field deficit, any documented visual field defect on confrontation testing or formal perimetry. Standardized neurological examinations were performed by trained neurologists in the inpatient setting.

### 2.4 Statistical analysis

Demographic and clinical characteristics were summarized using descriptive statistics. Cross-tabulation of demographic and clinical variables with tumor location was performed, and Pearson’s chi-square test was used to determine differences in proportions between groups. Age was analyzed both as a continuous variable (median, range) for descriptive purposes and dichotomized at 60 years for association analysis, since 60 years is a commonly used cut-off in surgical neuro-oncology and reflects the upper boundary of typical surgical candidacy and a clinically meaningful change in perioperative risk in our institution. Variables with substantial missing data, in particular optic nerve fundus examination findings (missing in 36.0% of patients owing to inconsistent ophthalmologic documentation), were excluded from association testing. Other clinical variables had <5% missing data and were analyzed using complete-case analysis. Multivariable logistic regression was not performed because key potential confounders—preoperative tumor size and volume, peritumoral edema, midline shift, multiplicity, and hormonal contraceptive exposure—were not consistently documented in the retrospective record and could not be reliably extracted. This is acknowledged as a limitation. A two-sided p-value <0.05 was considered statistically significant. All analyses were performed using IBM SPSS Statistics version 23 (IBM Corp., Armonk, NY, USA).

### 2.5 Ethical considerations

Ethical approval was granted by the Institutional Ethics Committee of Health Research, Faculty of Medicine, Universitas Indonesia, Jakarta, Indonesia (approval number: KET-996/UN2.F1/ETIK/PPM.00.02/2024) on 15 July 2024. The requirement for written informed consent was waived owing to the retrospective nature of the study.

## 3 Results

### 3.1 Demographic and clinical characteristics

A total of 531 patients with intracranial meningioma underwent tumor removal surgery between 2013 and 2023. The demographic and clinical characteristics of the cohort are summarized in **Table 1**. Women constituted 92.1% of subjects, with a median age of 45 years (range 18–82). The majority of tumors (77.2%) were located at the cranial base, with sphenoorbital meningioma being the most common cranial base subtype (43.5%). Within the non–skull base/convexity group (n=121), true convexity tumors accounted for the majority, with parasagittal and falcine meningiomas grouped under this category and intraventricular meningiomas (n=5) also included. Headache was the most frequent presenting symptom (80.4%), followed by cranial nerve deficits (75.5%).

**Table 1:** Demographic and clinical characteristics of subjects (n=531).

Variable	N (%)
Gender	
Male	42 (7.9)
Female	489 (92.1)
Age, years (median, range)	45 (18–82)
WHO Grade	
I	476 (89.6)
II/III	55 (10.4)
Location	
Non-skull base/convexity	121 (22.8)
True convexity, parasagittal, falcine	116 (21.8)
Intraventricular	5 (0.9)
Pineal (deep midline, non–skull base)*	1 (0.2)
Cranial base	410 (77.2)
Sphenoorbital	231 (43.5)
Sella-suprasellar	91 (17.1)
Posterior fossa	17 (3.2)
Olfactory	12 (2.3)
Cerebellopontine angle	21 (4.0)
Orbital/retro-orbital	7 (1.3)
Basal frontal/temporal	17 (3.2)
Cavernous sinus	0 (0)
Temporal fossa	1 (0.2)
Petroclival	6 (1.1)
Clinoidal	1 (0.2)
Simpson grade of resection	
Grade I	58 (10.9)
Grade II	94 (17.7)
Grade III	182 (34.3)
Grade IV	168 (31.6)
Grade V	16 (3.0)
Unknown	13 (2.4)
Decreased consciousness	
Yes	16 (3.0)
No	515 (97.0)
Headache	
Yes	427 (80.4)
No	104 (19.6)
Cranial nerve deficit (CN III–XII)	
Yes	401 (75.5)
No	130 (24.5)
Optic nerve abnormality (CN II)†	
Papilledema	76 (14.3)
Optic atrophy	177 (33.3)
Normal	87 (16.4)
Unknown	191 (36.0)
Motor deficit	
Yes	72 (13.6)
No	459 (86.4)
Sensory deficit	
Yes	18 (3.4)
No	513 (96.6)
Seizure	
Yes	53 (10.0)
No	478 (90.0)
Visual field deficit	
Yes	67 (12.6)
No	464 (87.4)

WHO, World Health Organization. CN, cranial nerve. *Pineal-region meningiomas were classified under the non–skull base group because of their supratentorial, deep midline location. †Optic nerve fundus findings were excluded from association testing in **Table 2** due to high proportion of missing data.

### 3.2 Association of demographic and clinical characteristics with tumor location

As shown in **Table 2**, several demographic and clinical variables were significantly associated with tumor location. Female gender (p=0.037), age <60 years (p<0.001), cranial nerve deficits (p<0.001), and visual field deficits (p=0.010) were significantly more frequent in cranial base meningiomas. Conversely, WHO grade II/III tumors (p<0.001), a higher proportion of Simpson grade I–II resection (p<0.001), decreased consciousness (p<0.001), headache (p=0.023), motor deficits (p<0.001), sensory deficits (p=0.009), and seizures (p<0.001) were significantly more frequent in non–skull base/convexity meningiomas. Optic nerve fundus abnormality (papilledema/optic atrophy) was not included in the association testing because of the high proportion of missing data (36.0%); descriptive findings are reported in **Table 1**.

**Table 2:** Association of demographic and clinical characteristics with tumor location (n=531).

Variable	Location		*p value*
	Cranial base	Non-skull base/convexity	
Gender			
Male	27 (6.6)	15 (12.4)	
Female	383 (93.4)	106 (87.6)	**0.037**
WHO Grade			
I	378 (92.2)	98 (81.0)	
II/III	32 (7.8)	23 (19.0)	**<0.001**
Age			
<60 years	395 (96.3)	104 (86.0)	
≥60 years	15 (3.7)	17 (14.0)	**<0.001**
Simpson grade			
Grade I-II	82 (20.0)	70 (57.9)	
Grade III-V	320 (77.3)	46 (40.4)	
Unknown	11 (2.7)	2 (1.7)	**<0.001**
Decreased consciousness			
Yes	3 (0.7)	13 (10.7)	
No	407 (99.3)	108 (89.3)	**<0.001**
Headache			
Yes	321 (78.3)	106 (87.6)	
No	89 (21.7)	15 (12.4)	**0.023**
Cranial nerve deficit (CN III-XII)			
Yes	349 (85.1)	52 (43.0)	
No	61 (14.9)	69 (57.0)	**<0.001**
Motor deficit			
Yes	26 (6.3)	46 (38.0)	
No	384 (93.7)	75 (62.0)	**<0.001**
Sensory deficit			
Yes	9 (2.2)	9 (7.4)	
No	401 (97.8)	112 (92.6)	**0.009**
Seizure			
Yes	25 (6.1)	28 (23.1)	
No	385 (93.9)	93 (76.9)	**<0.001**
Visual field deficit			
Yes	60 (14.6)	7 (5.8)	
No	350 (85.4)	114 (94.2)	**0.010**

Pearson’s chi-square test; two-sided p value <0.05 was considered statistically significant. WHO, World Health Organization. The non-skull base/convexity group includes true convexity, parasagittal, falcine, intraventricular, and pineal-region meningiomas. Optic nerve fundus findings were not included in this association table because 36.0% of records were missing. Simpson grade is shown as the conventional groupings I–II (gross total resection) and III–V (subtotal/partial resection). Bold values in the p value column indicate statistically significant associations (two-sided p<0.05, Pearson’s chi-square test).

## 4 Discussion

Tumor location in meningioma has important implications for treatment and prognosis. The aim of this study was to characterize the demographic and clinical differences between cranial base and non–skull base meningiomas in a large Indonesian surgical cohort. The female predominance observed in our cohort was striking: women accounted for 92.1% of subjects, yielding a female-to-male ratio of approximately 11:1, substantially higher than the 2–3:1 ratio reported in Western population-based cohorts (11). This extreme ratio should be interpreted with caution. Our cohort represents only patients who underwent surgical resection at a tertiary national referral center, and several non-biological factors may contribute, including: (i) referral bias toward a national center with established skull-base expertise, where women presenting with symptomatic skull-base lesions may be preferentially referred; (ii) institutional case-mix shaped by sub-specialty interest; (iii) sex-specific differences in healthcare-seeking behavior and access to surgical care; and (iv) potential under-detection of meningioma in men, who may be more likely to have small, asymptomatic tumors managed conservatively. Population-based data from Indonesia are limited, and our findings should not be extrapolated to incidence in the general population.

Biological factors may also contribute. Meningiomas are known to occur predominantly in women due to the role of progesterone and estrogen in their pathogenesis. In Indonesia, hormonal contraception is widely used among women of reproductive age; in 2023, 71.9% of women aged 10–54 used contraception, with more than 50% choosing hormonal methods (12). Hormonal contraceptive use is an established risk factor for meningioma (13), and progesterone receptor (PR) expression in meningiomas has been linked to female sex, premenopausal status, and exogenous hormone exposure (14). However, we did not collect data on hormonal contraceptive exposure in our cohort, and therefore any causal link between contraceptive use and the observed female predominance in our patients remains speculative and cannot be established by the present data. Other Indonesian single-center studies have reported similarly high female predominance (15, 16), but population-based confirmation is lacking.

Cranial base meningiomas accounted for 77.2% of our cohort, consistent with the epidemiological study by Champeaux-Depond et al., who reported cranial base meningioma in 73.9% of patients excluding spinal tumors (17). This high proportion likely reflects both the institutional referral pattern—our center is a national referral hospital with established skull-base surgery expertise—and selection bias inherent to a surgical cohort, since cranial base tumors are more often referred for resection because of symptom burden or proximity to critical structures. Cranial base location was significantly associated with female gender (p=0.037) in our cohort. Apra et al. demonstrated higher PR expression in women with sphenoorbital meningioma compared with men (9), and Malueka et al. reported that sphenoorbital meningioma is associated with hormonal contraceptive use (18).

Cranial base meningioma was associated with lower WHO grade (p<0.001) in our analysis, consistent with previous reports that grade 2 meningiomas are more commonly found outside the skull base (10). The mechanistic basis is incompletely understood, but possibilities include site-specific differences in cell of origin, microenvironment, and molecular drivers.

Clinical signs and symptoms also showed clear associations with tumor location. Decreased consciousness, headache, seizures, and motor and sensory deficits were associated with non–skull base tumors, while cranial nerve deficits and visual field deficits were associated with cranial base tumors. These findings are consistent with previously published studies. Wu et al. observed that seizures, motor and sensory deficits were more common in convexity meningiomas, while headache, anosmia, ocular, and cerebellar deficits were more common in cranial base meningiomas (19). The anatomical relationship is mechanistically plausible: cranial base tumors are more likely to compress cranial nerves and adjacent neurovascular structures, while non–skull base tumors compress pain-sensitive structures, generate epileptogenic microenvironments, and impair eloquent motor and sensory cortices through mass effect and surrounding vasogenic edema. It should be noted that presenting symptoms such as headache, seizure, and motor deficit may also depend strongly on tumor size, peritumoral edema, midline shift, and tumor multiplicity—none of which were systematically captured in the present dataset. These radiologic factors may attenuate or modify the location-symptom associations reported here.

Regarding extent of resection, Simpson grade I–II resection was significantly more common in non–skull base/convexity tumors, while Simpson grade III–V resection predominated in cranial base tumors. This pattern is expected and reflects the well-recognized anatomical and technical constraints of skull-base surgery: cranial base meningiomas frequently encase or are intimately adherent to critical neurovascular structures (cranial nerves, internal carotid and vertebrobasilar arteries, cavernous sinus, dural venous sinuses, and brainstem), which limits aggressive dural and bony resection. In contemporary skull-base practice, the priority has shifted from achieving Simpson grade I resection at all costs toward maximal safe resection with functional preservation, with planned residual tumor managed by observation or adjuvant stereotactic radiosurgery. Our findings are therefore better interpreted as reflecting surgical decision-making and anatomy rather than tumor biology alone.

### 4.1 Strengths and limitations

This study has several limitations. First, this is a single-center retrospective surgical cohort from a tertiary national referral hospital, and our findings reflect a selected population—only patients with symptomatic or otherwise operable tumors and only those referred to our institution. Incidentally discovered meningiomas managed conservatively are not represented, which limits the generalizability of our prevalence and demographic estimates to the underlying population. Referral bias may inflate the apparent proportion of skull-base tumors and the female predominance. Second, hormonal contraceptive exposure could not be reliably documented owing to incomplete follow-up data, and our interpretive comments on hormonal mechanisms therefore remain hypothesis-generating rather than confirmatory. Third, important radiologic variables—tumor size and volume, peritumoral edema, midline shift, and multiplicity—were not systematically captured and could not be included as covariates. Fourth, molecular profiling (PR/ER expression, somatic mutations, methylation) was not available for the cohort, which is a significant limitation given the recent shift toward integrated histo-molecular classification under the 2021 WHO criteria. Fifth, only univariate associations were tested; the absence of a multivariable model means that residual confounding among age, sex, WHO grade, and clinical presentation cannot be excluded, and the location-symptom associations reported here should be regarded as associative rather than independent. Sixth, optic nerve fundus examination data were missing for 36.0% of patients and could not be analyzed.

Despite these limitations, this study provides large-scale data on the association between presenting symptoms, tumor location, and histopathological grade in a Southeast Asian population, and demonstrates that clinical signs and symptoms can serve as useful early clues to tumor location. The disproportionately high female-to-male ratio observed in this and other Indonesian cohorts is a notable epidemiological signal that warrants confirmation in population-based, multicenter studies incorporating molecular and hormonal profiling.

### 4.2 Conclusion

Tumor location in meningioma is associated with specific clinical manifestations and histopathological grade. Cranial base tumors tend to present with cranial nerve–related and visual symptoms and are more often of WHO grade I, whereas non-skull base tumors are more likely to cause seizures, motor deficits, and decreased consciousness, and are more often of higher WHO grade. Recognition of these patterns may help clinicians prioritize diagnostic workup and surgical planning, particularly in resource-limited settings. Our findings should be interpreted within the limitations of a single-center retrospective surgical cohort, and future population-based studies incorporating radiologic, hormonal, and molecular variables are needed to confirm and extend these observations.

## Data Availability

The original contributions presented in the study are included in the article/supplementary material. Further inquiries can be directed to the corresponding author.
